# Preventive Cardiovascular Medication Prescribing Patterns in Menopausal Women

**DOI:** 10.1016/j.mayocpiqo.2026.100754

**Published:** 2026-09-01

**Authors:** Amanda Chang, Trisha Slehria, Jennah Johnson, Miquela Teske, Hannah O’Neill, Evelyn Ross-Shapiro, Wendy Fiordellisi

**Affiliations:** Department of Internal Medicine, University of Iowa, Iowa City, IA

## Abstract

**Objective:**

To characterize longitudinal prescribing patterns of preventive cardiovascular medications among women with nonsurgical menopause and women with surgical menopause to better identify potential differences in care.

**Patients and Methods:**

We conducted a retrospective multicenter review using TriNetX from January 27, 2006, to January 27, 2026 (TriNetX analyzes up to 20 years of data before the analysis date). Nonsurgical and surgical menopause patients were compared regarding statin, antihypertensive, and diabetes medication prescription rates over 5 years. Prescribing patterns were additionally analyzed based on medication classes.

**Results:**

A total of 690,799 nonsurgical menopause patients and 28,473 surgical menopause patients were queried. Nonsurgical menopausal women consistently had lower rates of statin (6.0%), antihypertensive (17.1%), and diabetes medications (7.7%) over 5 years than surgical menopause patients (*P*<.0001).

**Conclusion:**

Surgical menopause patients had higher rates of statin, antihypertensive, and diabetes prescriptions than nonsurgical menopause patients matched by age and comorbidities. Providers should be cognizant of the interplay between menopause status and cardiovascular risk when prescribing preventive cardiovascular medications.

Cardiovascular disease (CVD) remains the leading cause of morbidity and mortality among women, with risk accelerating across the menopause transition and persisting into menopause.^1^^,^^2^ The menopause transition, also commonly referred to as perimenopause, occurs during a period with increased variability in menstrual cycle length because of the increasing rate of ovarian follicle depletion, most commonly lasting 4 to 5 years. Nonsurgical (natural) menopause occurs after 12 months of amenorrhea in women aged 45 years and older, with the median age ranging between 51 and 52 years in North America.^3^

Significant cardiometabolic changes associated with ovarian aging and the menopause transition include adverse lipid profiles, carotid atherosclerosis, vascular stiffness, and increased adiposity.^3^ Menopausal women have higher rates of CVD, and this effect appears particularly pronounced among women who experienced surgical menopause.^4^^,^^5^ Women who undergo surgical menopause, particularly bilateral oophorectomy performed before the age of nonsurgical menopause, demonstrate increased rates of CVD, coronary artery disease, and cardiovascular mortality compared with those who do not.^4^

Furthermore, although the menopause transition is associated with a well-established increase in cardiovascular risk, prior studies consistently show that men are more likely than women to receive statins and other guideline-directed preventive cardiovascular medications, despite the menopause transition representing a critical window for intervention.^1^^,^^2^^,^^6^ Additionally, menopause status is not consistently incorporated into routine quantitative cardiovascular risk assessment tools.^1^^,^^7^

Sex-based differences in preventive pharmacotherapy have been documented across multiple cardiovascular risk domains. However, these analyses have largely focused on sex differences broadly, rather than on menopause-defined subgroups of women.^8^^,^^9^

There is a paucity of data comparing CVD preventive medication use in women with nonsurgical versus in those with surgical menopause.^10^ Furthermore, longitudinal changes in prescribing patterns stratified by menopause status have not been well characterized in the existing literature. We aim to better characterize the differences in longitudinal prescribing patterns of cardiovascular preventive medications for hyperlipidemia, hypertension, and diabetes between surgical and nonsurgical menopause patients.

## Patients and Methods

### Study Design

We conducted a retrospective observational study comparing the prescription outcomes of women who experienced surgical (bilateral oophorectomy) menopause and nonsurgical (natural) menopause. A flowchart of the study design is present in the Supplemental Figure 1 (available online at http://www.mcpiqojournal.org).

### Data Source

Data were obtained from the TriNetX research network (TriNetX), which consolidates updated information from 110 large health care organizations with over 120 million electronic medical patient records. Information includes demographic characteristics, diagnoses, procedures, laboratory values, medications, and outcomes. Diagnoses are classified via the International Classification of Diseases, Tenth Revision (ICD-10). As a federated network, TriNetX has obtained a Western Institutional Review Board (IRB) waiver and is Health Insurance Portability and Accountability Act compliant. This study was additionally reviewed by the University of Iowa IRB and was deemed exempt from formal review (IRB 202601210).

### Study Population

Records of patients aged between 40 and 60 years, reviewed from January 27, 2006, to January 27, 2026, were included in the study. Female patients were separated into surgical or nonsurgical menopause groups given disparate CVD risk. Female nonsurgical menopause patients were included if they had a diagnosis of menopause or symptomatic premature menopause. Surgical menopause patients were classified if they had bilateral oophorectomy. The index event was the first diagnosis of menopause/premature menopause in the nonsurgical group, while the acquired absence of bilateral ovaries was the index event for the surgical group. Patients were excluded from all groups if they (1) had prior CVD (ischemic heart disease, prior revascularization, heart failure, atrial fibrillation/flutter, stroke, peripheral artery disease, aortic stenosis, mitral regurgitation, and venous thromboembolism), (2) were on statin, antihypertensive, or diabetes medications previously, or (3) had a contraindication to statins. Relevant ICD-10 codes can be reviewed in the Supplemental Tables 1 and 2 (available online at http://www.mcpiqojournal.org).

### Propensity Score Matching

For each cohort, propensity score matching was conducted via built-in functions through TriNetX to generate 1:1 matched cohorts based on greedy nearest neighbor matching with default caliper width of 0.1 pooled SDs and the following confounders: age at index, race, and comorbidities (diabetes, hypertension, chronic kidney disease (CKD), body mass index, and hyperlipidemia). Cohorts were matched without replacement. Propensity score density graphs before and after matching are available in Supplemental Figure 2 (available online at http://www.mcpiqojournal.org).

### Outcomes

All patients were followed up for 5 years, with interval results at 1 month, 3 months, 1 year, 3 years, and 5 years. The primary outcome was rate of prescriptions for statins, antihypertensive medications, and diabetes medications. Secondary outcomes were timing of specific antihypertensive medication prescriptions and specific diabetes medication prescriptions.

### Statistical Analyses

Statistical analysis was performed using built-in TriNetX functions. Baseline cohort characteristics were presented as a mean with SD for continuous variables and a number and percentage for categorical variables. Statistical differences for continuous variables were evaluated using Student *t* test, whereas categorical variables were compared via χ^2^ tests. Propensity score matching used a standardized mean difference of less than 0.1, indicating adequate matching between the 2 groups. Differences in outcomes were assessed using odds ratios (ORs) and 95% CIs. A *P* value of .05 or less was considered statistically significant.

## Results

### Baseline Characteristics

A total of 690,799 nonsurgical menopause patients with median age of 48.9 ± 5.2 years and 28,473 surgical menopause patients with median age of 46.4 ± 6.1 years were identified for the study. Of the nonsurgical menopause patients identified, 40.1% (313,524 patients) had essential hypertension, 18.1% (141,656 patients) had hyperlipidemia, 9.6% (75,387 patients) had CKD, and 33.5% (261,799 patients) had diabetes. Of the surgical menopause patients identified, 39.48% (4739 patients) had essential hypertension, 23.4% (2255 patients) had hyperlipidemia, 12.4% (1194 patients) had CKD, and 31.7% (3052 patients) had diabetes. These results are summarized in Table 1.

**Table 1:** Baseline and Propensity Score–Matched Characteristics of Patients

Category	Variable	Nonsurgical menopause	Surgical menopause	*P* (nonsurgical vs surgical menopause)	SMD (nonsurgical vs surgical menopause)
Demographic characteristics	N	690,799	28,473		—
	Age at index (y)	48.9 ± 5.2	46.4 ± 6.1	<.0001	—
	White	484,757 (70.2)	20,533 (72.1)	<.0001	—
	BMI (kg/m^2^)	28.1 ± 6.6	29.8 ± 7.6	<.0001	—
Comorbidities	Essential hypertension	313,524 (40.1)	4739 (39.48)	<.0001	—
	Hyperlipidemia	141,656 (18.1)	2255 (23.4)	.90	—
	Chronic kidney disease	75,387 (9.6)	1194 (12.4)	<.0001	—
	Diabetes	261,799 (33.5)	3052 (31.7)	<.0001	—
Demographic characteristics	Total N	28,472	28,472		—
	Age at index (y)	46.4 ± 6.1	46.4 ± 6.1	.93	0.0007
	White	20,532 (72.1)	20,532 (72.1)	>.99	<0.0001
	BMI (kg/m^2^)	29.8 ± 7.6	29.8 ± 7.6	.96	0.0004
Comorbidities	Essential hypertension	2795 (9.8)	2797 (9.8)	.98	0.0002
	Hyperlipidemia	1944 (6.8)	1944 (6.8)	>.99	<0.0001
	Chronic kidney disease	204 (0.72)	221 (0.78)	.84	0.0016
	Diabetes	1086 (3.8)	1095 (3.8)	.41	0.0069

Values are given as mean ± SD or n (%).

BMI, body mass index; SMD, standardized mean difference.

Propensity score matching resulted in 2 cohorts with minimal differences. The cohorts consisted of 28,472 nonsurgical menopause patients and 28,472 surgical menopause patients, with a median age of 46.4 ± 6.1 years. In both groups, 9.8% of patients had essential hypertension, and 6.8% had hyperlipidemia. Nonsurgical menopause patients and surgical menopause had CKD rates of 0.72% and 0.78%, respectively. Both nonsurgical and surgical menopause patients had a rate of 3.8% for diabetes. These results are summarized in Table 1.

### Statin Prescription Outcomes

**Figure 1:**
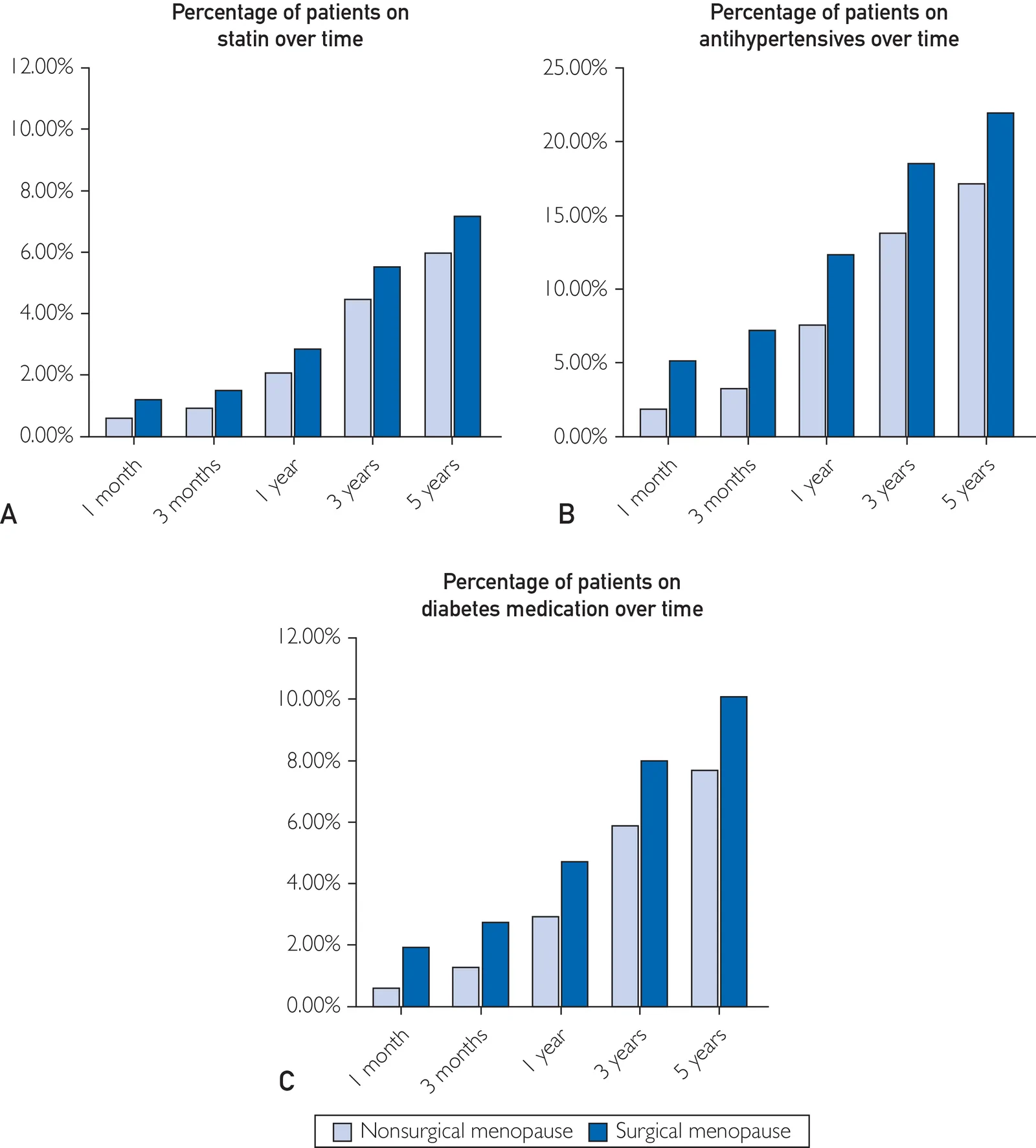
Prescription rates of statins, antihypertensives, and diabetes medications over time for nonsurgical menopause and surgical menopause patients. Prescription rates for all medication categories increased over time for both groups. (A) Surgical menopause patients had consistently higher rates of statin prescription than nonsurgical menopause patients. (B) Surgical menopause patients consistently had higher rates of antihypertensive prescriptions than nonsurgical menopause patients. (C) Surgical menopause patients had the highest rate of diabetes medication prescription over 5 years.

**Figure 2:**
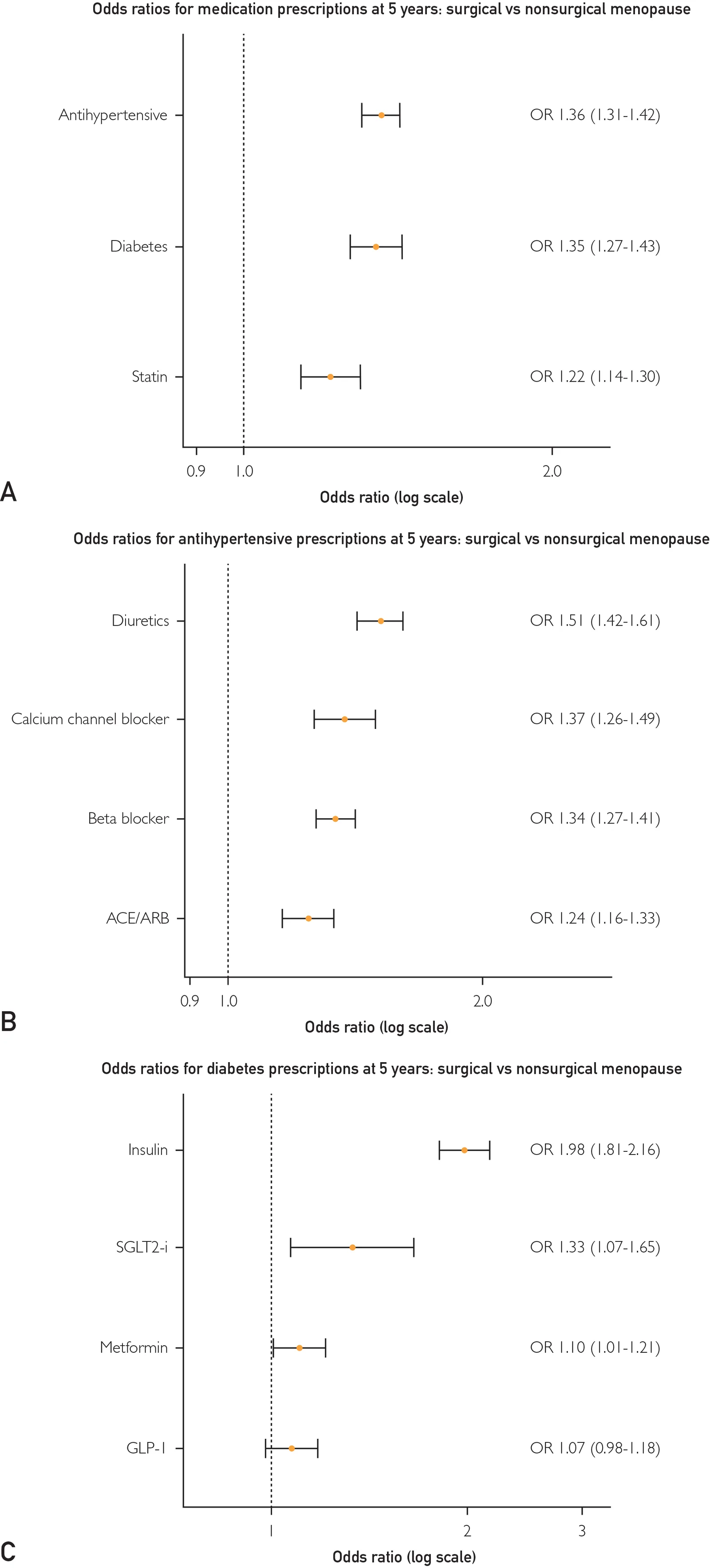
Forest plots for medication prescriptions at 5 years. (A) Odds ratios for overall medications for surgical versus nonsurgical menopause patients. (B) Odds ratios for antihypertensive subclasses prescriptions for surgical versus nonsurgical menopause patients. (C) Odds ratios for diabetes prescriptions for surgical versus nonsurgical menopause patients. GLP, glucagon-like peptide; SGLT2-i, sodium-glucose cotransporter-2 inhibitor.

Statin prescription rates increased over time for both groups (Figure 1A). At 5 years, statin prescription rates were 7.2% for surgical menopause patients and 6.0% for nonsurgical menopause patients (OR, 1.22; 95% CI, 1.14-1.30) (Figure 2A). Nonsurgical menopause patients had significantly lower rates of statin prescriptions than surgical menopause patients (*P*<.0001) (Table 2).

**Table 2:** Overall Medication Prescription Rates Over Time

Medication category	Period	Nonsurgical menopause (%)	Surgical menopause (%)	*P* (nonsurgical vs surgical menopause)
Overall medication				
Statin	1 mo	0.54	1.16	<.0001
	3 mo	0.91	1.49	<.0001
	1 y	2.08	2.81	<.0001
	3 y	4.43	5.50	<.0001
	5 y	5.97	7.17	<.0001
Antihypertensive	1 mo	1.84	5.05	<.0001
	3 mo	3.19	7.21	<.0001
	1 y	7.53	12.27	<.0001
	3 y	13.72	18.53	<.0001
	5 y	17.08	21.91	<.0001
Diabetes medications	1 mo	0.64	1.92	<.0001
	3 mo	1.28	2.74	<.0001
	1 y	2.98	4.77	<.0001
	3 y	5.88	8.00	<.0001
	5 y	7.67	10.06	<.0001
Antihypertensive medication				
ACE/ARB	1 mo	0.48	1.24	<.0001
	3 mo	0.90	1.71	<.0001
	1 y	2.18	3.19	<.0001
	3 y	4.04	5.23	<.0001
	5 y	5.34	6.55	<.0001
β-Blockers	1 mo	0.66	2.54	<.0001
	3 mo	1.57	3.67	<.0001
	1 y	3.76	6.45	<.0001
	3 y	7.15	9.98	<.0001
	5 y	9.25	12.01	<.0001
Calcium channel blockers	1 mo	0.32	0.99	<.0001
	3 mo	0.52	1.34	<.0001
	1 y	1.32	2.31	<.0001
	3 y	2.63	3.93	<.0001
	5 y	3.55	4.81	<.0001
Diuretics	1 mo	0.44	2.11	<.0001
	3 mo	0.96	2.95	<.0001
	1 y	2.49	4.97	<.0001
	3 y	4.99	7.75	<.0001
	5 y	6.36	9.32	<.0001
Diabetes medications				
Metformin	1 mo	0.27	0.39	.0085
	3 mo	0.51	0.63	.0746
	1 y	1.16	1.37	.0222
	3 y	2.30	2.66	.0055
	5 y	3.20	3.52	.0343
Insulin	1 mo	0.21	1.55	<.0001
	3 mo	0.43	2.04	<.0001
	1 y	0.99	3.06	<.0001
	3 y	2.07	4.50	<.0001
	5 y	2.75	5.30	<.0001
SGLT2-i	1 mo	n/a	0.06	n/a
	3 mo	0.06	0.09	.17
	1 y	0.12	0.22	.0046
	3 y	0.31	0.44	.0111
	5 y	0.50	0.67	.0098
GLP-1	1 mo	0.18	0.13	.17
	3 mo	0.33	0.45	.0222
	1 y	1.08	0.93	.0852
	3 y	2.28	2.27	.93
	5 y	3.23	3.46	.13

ACE, angiotensin-converting enzyme; ARB, angiotensin receptor blocker; GLP, glucagon-like peptide; SGLT2-i, sodium-glucose cotransporter-2 inhibitor.

### Antihypertensive Prescription Outcomes

Antihypertensive prescriptions increased in both groups over time (Figure 1B). Overall antihypertensive prescription rates at 5 years were 21.9% for surgical menopause patients and 17.1% for nonsurgical menopause patients (OR, 1.36; 95% CI, 1.31-1.42) (Figures 1B and 2A). Surgical menopause patients had significantly higher rates of antihypertensive prescriptions than nonsurgical menopause patients at all periods (*P*<.0001) (Table 2).

**Figure 3:**
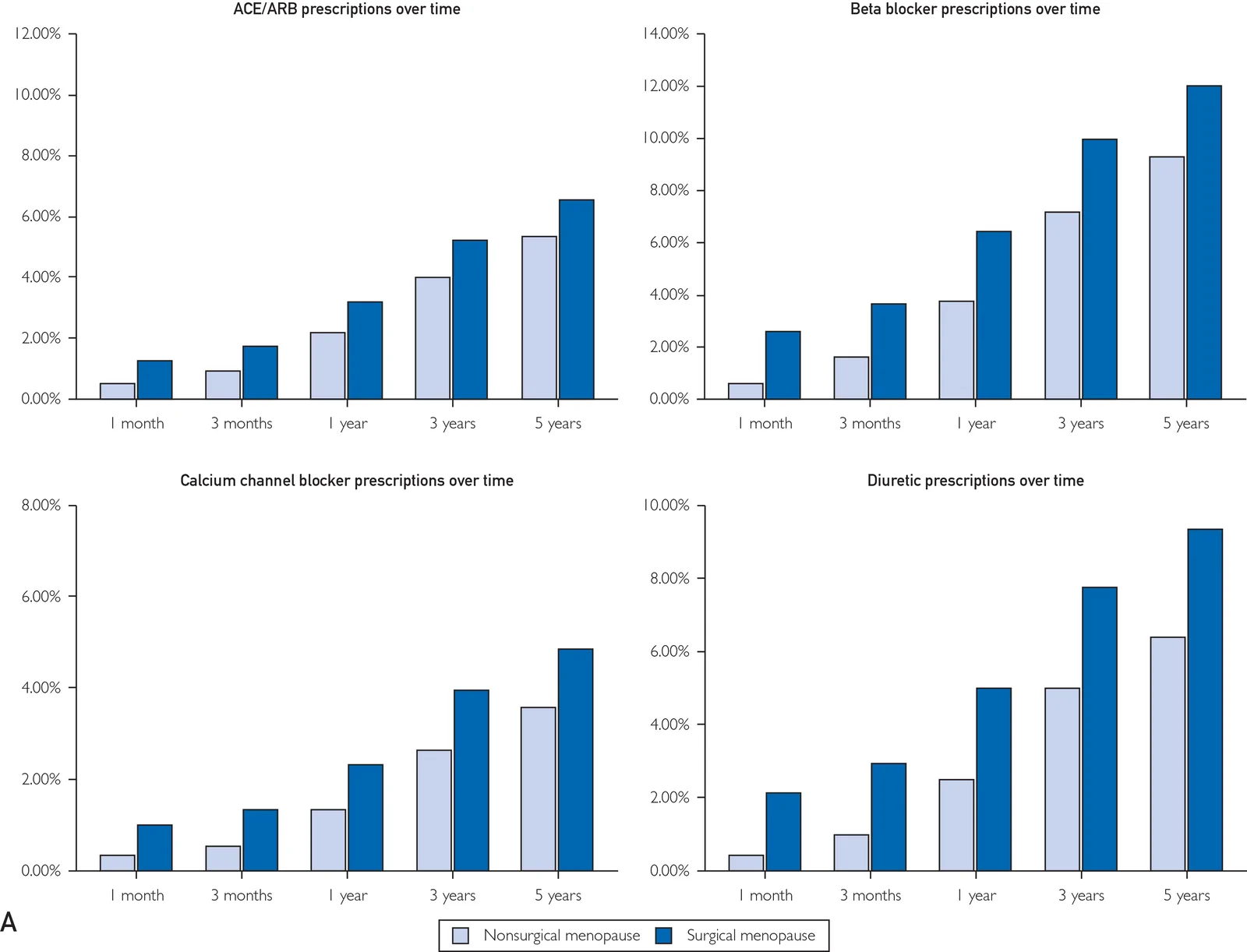

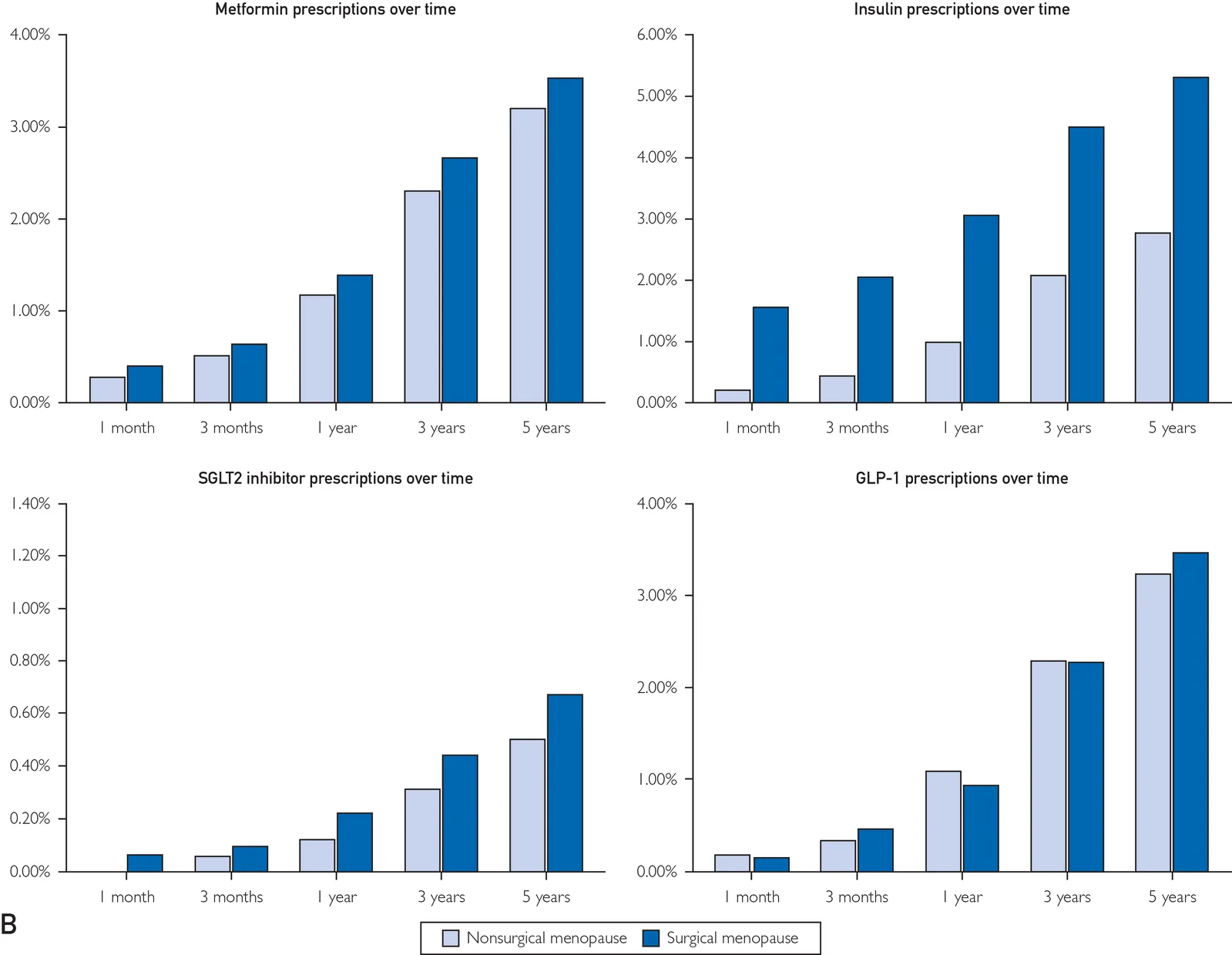
Prescription rates of antihypertensive and diabetes medications over time by subclasses. (A) Surgical menopause patients had significantly higher rates of all antihypertensive subclass (ACE/ARB, β-blocker, calcium channel blocker, and diuretics) prescriptions throughout all periods than nonsurgical menopause patients. (B) Surgical menopause patients had higher rates of all metformin, insulin, and SGLT2-i prescriptions in all periods. Nonsurgical menopause patients had higher rates of GLP-1 prescriptions at 1 and 3 years. After 5 years, surgical menopause patients had higher rates of GLP-1 prescriptions. ACE, angiotensin-converting enzyme; ARB, angiotensin receptor blocker; GLP, glucagon-like peptide; SGLT2-i, sodium-glucose cotransporter-2 inhibitor.

Surgical menopause patients had significantly higher rates of all antihypertensive class prescriptions (angiotensin-converting enzyme inhibitors/angiotensin receptor blocker, β-blockers, calcium channel blockers, and diuretics) than nonsurgical menopause patients at all periods (*P*<.0001) (Figure 3A). At 5 years, the rates of antihypertensive class prescriptions were as follows between surgical menopause and nonsurgical menopause patients, respectively: angiotensin-converting enzyme inhibitors/angiotensin receptor blocker, 6.6% versus 5.3% (OR, 1.24; 95% CI, 1.16-1.33); β-blockers, 10.3% versus 9.3% (OR, 1.33; 95% CI, 1.27-1.41); calcium channel blockers, 4.8% versus 3.6% (OR, 1.37; 95% CI, 1.26-1.49); and diuretics, 9.3% versus 6.4% (OR, 1.51; 95% CI, 1.42-1.61) (Figure 2B). These results are summarized in Table 2.

### Diabetes Medication Prescription Outcomes

Both groups had an increase in diabetes medication prescriptions over time (Figure 1C). Diabetes medication prescription rates at 5 years were 10.1% for surgical menopause patients and 7.7% for nonsurgical menopause patients (OR, 1.35; 95% CI, 1.27-1.43) (Figures 1C and 2A). Surgical menopause patients had significantly higher rates of diabetes medication prescriptions than nonsurgical menopause patients at all periods (*P*<.0001) and male patients at 3 and 5 years (*P*<.0001) as reviewed in in Table 2.

Compared with nonsurgical menopause patients, surgical menopause patients had significantly higher rates of insulin prescriptions over all periods (*P*<.0001). Surgical menopause patients had higher rates of metformin, insulin, and sodium-glucose cotransporter-2 inhibitor (SGLT2-i) prescriptions, although there were no significant differences in glucagon-like peptide (GLP)-1 prescriptions. Specifically, at 5 years, diabetes prescription rates of surgical menopause and nonsurgical menopause patients were as follows, respectively: metformin, 3.5% versus 3.2% (OR, 1.10; 95% CI, 1.01-1.21); insulin, 5.3% versus 2.8% (OR, 1.98; 95% CI, 1.81-2.16); SGLT2-I, 0.67% versus 0.50% (OR, 1.33; 95% CI, 1.07-1.65); and GLP-1 3.5% versus 3.2% (OR, 1.07; 95% CI, 0.98-1.17) (Figures 2C and 3B). These results are summarized in Table 2.

## Discussion

In this large, propensity score–matched retrospective analysis, we characterized longitudinal preventive cardiometabolic medication prescribing patterns among women with surgical and nonsurgical menopause over a 5-year follow-up period. Nonsurgical menopause patients had consistently lower rates of statin, antihypertensive, and diabetes medication prescriptions than surgical menopause patients, despite comparable propensity score–matched baseline comorbidity profiles. These differences were apparent as early as 1 month following the index event and persisted over the course of 5 years. Taken together, these findings suggest that menopause subtype may be a meaningful factor in cardiovascular risk assessment, further highlighting the potential value of incorporating menopause-specific characteristics into preventive care for midlife women.

The lower rates of statin prescribing observed among nonsurgical menopause patients compared with surgical menopause patients are particularly notable. Statins are primary medications for primary prevention of atherosclerotic CVD and their underutilization in women has been previously documented.^6^ Further, the disparity in our study may reflect greater clinician recognition of CVD risk following surgical menopause, as this is often perceived as a more abrupt and severe endocrinologic transition, and there is evidence that surgical menopause confers elevated risk when compared with nonsurgical menopause.^5^ Given the more gradual clinical course, nonsurgical menopause CVD risk may be less readily appreciated by providers, although it is important to recognize that risk increases across the menopausal transition regardless of menopause etiology.^4^ This has important clinical implications in that nonsurgical constitutes most of menopausal women and may represent a systematically underrecognized opportunity for primary cardiovascular prevention.

Surgical menopause patients also showed higher rates of antihypertensive prescriptions over time than nonsurgical menopausal women (Figure 1B). Increased use of antihypertensives among the surgical menopausal group may be supported by prior literature demonstrating that oophorectomy is associated with increases in blood pressure and peripheral vascular resistance, suggesting a role of ovarian hormone loss in blood pressure regulation.^11^ Additionally, epidemiologic studies show that prevalence of hypertension rises after menopause, with blood pressure typically increasing more steeply in aging women.^12^^,^^13^ This is consistent with the notion that clinically apparent hypertension may not emerge until 5 to 10 years following menopause.^14^^,^^15^ This aligns with our findings that antihypertensive prescriptions in the surgical menopause cohort increased over time and were highest at 5 years following menopause (Figure 2).

Subgroup analysis of antihypertensive medication classes revealed that the overall difference between the surgical and nonsurgical menopausal groups was driven primarily by higher use of β-blockers and diuretics (Figure 3A). Prior literature similarly describes sex-based differences in antihypertensive class prescribing, but comparatively little data exist characterizing antihypertensive class selection within menopause subtypes specifically.^16^^,^^17^ In menopausal women, diuretics have been associated with effective blood pressure control as monotherapy.^18^ The higher diuretic use in the surgical group may reflect the increased salt sensitivity documented following oophorectomy, as ovarian hormone loss appears to promote sodium retention and hypertension.^19^ Additionally, higher β-blocker use in the surgical menopause cohort may reflect comorbidity patterns such as palpitations, arrhythmias other than atrial fibrillation or atrial flutter, anxiety, or variceal prophylaxis in cirrhosis, which were not specifically analyzed in this data set and should be explored in future works.^20^, ^21^, ^22^

Diabetes medication prescription rates were also higher among surgical menopause patients; with the differences most pronounced for insulin and SGLT2-i use. One potential explanation is that abrupt ovarian hormone loss may accelerate adverse metabolic changes, including increase in adiposity and insulin resistance, contributing to greater downstream diabetes burden and treatment intensification in surgical menopausal women.^23^, ^24^, ^25^, ^26^

The higher insulin prescription rates in the surgical menopause group (OR, 1.98; 95% CI, 1.81-2.16) are particularly notable given that baseline diabetes prevalence was equivalent between matched cohorts (∼3.8% in each group). This potentially suggests worsening glycemic control rather than greater baseline diabetes burden. Additionally, because the analysis did not differentiate between type 1 and type 2 diabetes, it is possible that a higher prevalence of type 1 diabetes, which requires insulin as its primary treatment and disproportionately affects younger patients, may have contributed to the elevated insulin prescription rates observed in the surgical menopause cohort, independent of glycemic control and oophorectomy-related metabolic changes.

By contrast, GLP-1 receptor agonist prescriptions did not differ significantly between surgical and nonsurgical menopause patients at 5 years (OR, 1.07; 95% CI, 0.98-1.17). This null finding is noteworthy given the expansion of GLP-1 prescribing across cardiometabolic indications. Because GLP-1 receptor agonists are increasingly prescribed for obesity management in addition to type 2 diabetes, the observed prescriptions may not exclusively reflect glycemic treatment. Future analyses with indication-level prescription data would help clarify whether GLP-1–prescribing patterns differ between menopause subtypes specifically for diabetes versus obesity or weight management indications.

Menopausal hormone therapy (MHT) was not an exclusion criterion in this study. This warrants acknowledgment, as MHT, which is typically prescribed for vasomotor symptoms in menopause transition or during menopause itself, has effects on cardiometabolic risk and may influence both cardiovascular risk burden and prescribing patterns. MHT use may attenuate some of the cardiometabolic consequences of ovarian hormone loss, potentially confounding the observed differences in preventive medication prescription between surgical and nonsurgical menopause groups. Analyses incorporating MHT use as a covariate, either as a dichotomous variable or accounting for the duration of use, would provide a more complete understanding of the drivers of prescribing differences in this population and represent an important direction for future works.

Our study has characterized key differences in prescribing patterns between nonsurgical and surgical menopause patients. Future studies should build upon our findings and optimize personalized CVD risk management in menopausal patients. Potential avenues of further investigation include inclusion and risk stratification of perimenopausal women, prospective studies on the impact of newer diabetes medications (SGLT2-i and GLP-1) on CVD outcomes in menopausal patients, and evaluation of the CVD impact of MHT therapy with concurrent preventive medications.

### Limitations

This study has several important limitations. First, although TriNetX is an expansive multi–health care electronic database, diagnoses are limited by the accuracy of ICD-10 coding. As a result, the number of menopausal women may be underestimated due to inconsistent coding or misclassification. More specially, a key limitation is the undercoding of nonsurgical menopause in electronic health record data sets. Nonsurgical menopause is a physiologic transition that frequently occurs without a discrete clinical encounter or assigned diagnostic code. Thus, many women may not receive a formal ICD designation. As a result, the nonsurgical menopause group in this study may represent a subset of women who sought care specifically for menopausal symptoms or related conditions, rather than a broader population. This may limit the generalizability of our findings to the wider nonsurgical menopause population and could influence the observed prescribing patterns because women who are more engaged with the health care system for menopause related concerns may differ in their cardiometabolic risk profiles and medication use from those who are not.

Of note, there is no biological equivalent to menopause in males, therefore making physiologic male control cohort less feasible on the TriNetX database. We did attempt analysis using ambulatory visit rather than menopause as an index for a potential control group, and these results are available in Supplemental Tables 2 and 3 (available online at http://www.mcpiqojournal.org). We also did not match or adjust for specific vital signs, such as blood pressure, or laboratory values, including lipid measurements, blood glucose, or hemoglobin A1c. As a result, medication prescribing could not be assessed in relation to individual blood pressure values or biomarker-based treatment thresholds, which may limit interpretation of prescribing patterns.

Additionally, this study evaluated prescription patterns rather than guideline-based treatment eligibility. Contemporary risk calculators used to guide preventive therapy do not explicitly incorporate menopause status or menopause subtype, and therefore, observed differences in prescribing may reflect limitations of current risk estimation frameworks rather than clinician practice alone. TriNetX also lacks built-in CVD risk calculators to evaluate concordance between CVD risk and statin prescriptions, thereby limiting our results.

Regarding prescriptions, we evaluated prescriptions recorded in the electronic health record rather than actual medication fill history and adherence among patients. Our outcomes therefore reflect clinician-prescribing behavior rather than true medication exposure. Sex- and menopause related differences in medication acceptance, discontinuation, or intolerance could not be accounted for. Additionally, SGLT2-i and GLP-1 medications are relatively newer therapies, and lower prescription rates may reflect barriers to use such as cost, patient preference, or provider familiarity. Furthermore, SGLT2-i and GLP-1 medications may be prescribed for a variety of metabolic indications beyond diabetes management.

Additionally, some medications have additional indications other than our broad classifications. For example, β-blockers and diuretics may be prescribed for conditions other than hypertension, including arrhythmias, heart failure, and symptom management (such as β-blockers for anxiety). However, we did exclude patients with prior CVD (ischemic heart disease, prior revascularization, heart failure, atrial fibrillation/flutter, stroke, peripheral artery disease, aortic stenosis, mitral regurgitation, and venous thromboembolism) to mitigate the lack of granularity.

## Conclusion

Nonsurgical menopausal women had significantly lower rates of statin, antihypertensive, and diabetes medication prescriptions than surgical menopausal women. Regardless of etiology, the menopausal transition represents a critical window during which cardiometabolic risk accelerates, and preventive intervention may be impactful. These findings highlight important differences in preventive cardiovascular prescribing patterns across menopause subtypes, which underscore the need for personalized cardiovascular risk assessment strategies in midlife women.

## Potential Competing Interests

The authors report no competing interests.

## Ethics Statement

Data were obtained from the TriNetX research network (TriNetX), which consolidates updated information from 110 large health care organizations with over 120 million electronic medical patient records. As a federated network, TriNetX has obtained a Western Institutional Review Board (IRB) waiver and is Health Insurance Portability and Accountability Act compliant. This study was additionally reviewed by the University of Iowa IRB and was deemed exempt from formal review (IRB 202601210).
