# Supplementary material for: Preventive Cardiovascular Medication Prescribing Patterns in Menopausal Women
Source: Mayo Clin Proc Innov Qual Outcomes. 2026 Sep 1;10(5):100754. doi: 10.1016/j.mayocpiqo.2026.100754 (PMC13563236; doi:10.1016/j.mayocpiqo.2026.100754)
Supplement: Supplementary file 1 [file mmc1.docx]

March 11, 2026

Re: JADV013026-0282, Preventative Cardiovascular Medication Prescribing Patterns in Menopausal Women


Dear Dr. Slehria:

Thank you for submitting your manuscript to JACC: Advances. Unfortunately, after careful consideration by the editors and by expert external reviewers, the consensus is that this manuscript does not generate the degree of priority for publication. The comments of the reviewers are enclosed for your information (below).

We recognize the thought and effort that went into your work. However, we are only able to publish very few of the manuscripts we receive, and therefore, must decline many of considerable merit.

Again, thank you for your interest in the Journal, and we look forward to reviewing other submissions from you in the future.

Sincerely,

Agnes Koczo
Associate Editor
JACC: Advances


Candice Silversides, MD
Editor-in-Chief
JACC: Advances
Heart House,
2400 N Street, NW
Washington, DC 20037
Telephone: 202-375-6136
Fax: 202-375-6819
Email: jaccadvances@acc.org
