# Supplementary material for: Preventive Cardiovascular Medication Prescribing Patterns in Menopausal Women
Source: Mayo Clin Proc Innov Qual Outcomes. 2026 Sep 1;10(5):100754. doi: 10.1016/j.mayocpiqo.2026.100754 (PMC13563236; doi:10.1016/j.mayocpiqo.2026.100754)
Supplement: Supplementary file 2 [file mmc2.docx]

**Supplementary Figure 1: Study design**

**Supplementary Figure 2: Propensity Score Density Function Before and After Matching**

**Supplementary Table 1: ICD-10 Codes for Diagnoses and Comorbidities**

| Diagnosis | ICD-10 Code |
| --- | --- |
| Menopause and other perimenopausal disorders | N95 |
| Symptomatic premature menopause | E28.310 |
| Asymptomatic premature menopause | E28.319 |
| Asymptomatic menopausal state | Z78.0 |
| Acquired absence of ovaries, bilateral | Z90.722 |
|  |  |
| Ischemic heart diseases | I20-I25 |
| Diabetes mellitus | E08-E13 |
| Essential (primary) hypertension | I10 |
| Chronic Kidney Disease | N18 |
| Heart failure | I50 |
| Atrial fibrillation and flutter | I48 |
| Cerebral infarction | I63 |
| Peripheral vascular disease | I70-I79 |
| Pulmonary embolism | I26 |
| Other venous embolism and thrombosis | I82 |
| Nonrheumatic aortic valve diseases | I35 |
| Rheumatic aortic valve diseases | I06 |
| Nonrheumatic mitral valve diseases | I34 |
| Rheumatic mitral valve diseases | I05 |
| Presence of cardiac and vascular implants and grafts | Z95 |
|  |  |
| Toxic liver disease | K71 |
| Hepatic failure | K72 |
| Drug-induced myopathy | G72.0 |

**Supplementary Table 2: TriNetX Codes for Medications**

| Medications | TriNetX Code |
| --- | --- |
| Statins (HMG CoA reductase inhibitors) | C10AA |
| Diuretics | C03 |
| Beta blockers | C07 |
| Calcium channel blockers | C08 |
| ACE inhibitors/ARBs | C09 |
| SGLT2 inhibitors | C09 |
| Metformin | A10BK |
| Insulin | A10A |
| GLP-1 | A10BJ |

**Supplementary Table 2A: Baseline Characteristics of Patients (Including Male Patients)**

| ***CATEGORY*** | *Variable* | *Nonsurgical menopause* | *Surgical menopause* | *Men* | *p-value (Nonsurgical vs Surgical menopause)* | *p-value (Surgical menopause vs Men)* |
| --- | --- | --- | --- | --- | --- | --- |
| ***DEMOGRAPHICS*** | Total N | 690,799 | 28,473 | 12,375,303 |  |  |
|  | Age at index (years), mean ± SD | 48.9 ± 5.2 | 46.4 ± 6.1 | 41.7 ± 7.8 | <0.0001 | <0.0001 |
|  | White:  N (%) | 484,757 (70.2%) | 20,533 (72.1%) | 5,798,109 (50.5%) | <0.0001 | <0.0001 |
|  | BMI (kg/m^2^), mean ± SD | 28.1 ± 6.6 | 29.8 ± 7.6 | 29.0 ± 6.2 | <0.0001 | <0.0001 |
| ***COMORBIDITIES*** | Essential hypertension | 313,524 (40.1%) | 4739 (39.48%) | 165,513 (1.4%) | <0.0001 | <0.0001 |
|  | Hyperlipidemia | 141,656 (18.1%) | 2255 (23.4%) | 77,348 (0.67%) | 0.9 | <0.0001 |
|  | Chronic kidney disease | 75,387 (9.6%) | 1194 (12.4%) | 16,343 (0.14%) | <0.0001 | <0.0001 |
|  | Diabetes | 261,799 (33.5%) | 3052 (31.7%) | 63,122 (0.55%) | <0.0001 | <0.0001 |

**Supplementary Table 2B: Propensity Matched Characteristics (Including Male Patients)**

| ***CATEGORY*** | *Variable* | *Nonsurgical menopause* | | *Surgical menopause* | *Men* | *p-value (Nonsurgical vs surgical menopause)* | *SMD  (Nonsurgical vs Surgical menopause)* | *p-value (surgical menopause vs men)* | *SMD(surgical menopause vs men)* |
| --- | --- | --- | --- | --- | --- | --- | --- | --- | --- |
| ***DEMOGRAPHICS*** | Total N | 28,472 | | 28,472 | 28,468 |  |  |  |  |
|  | Age at index (years), mean ± SD | | 46.4 ± 6.1 | 46.4 ± 6.1 | 46.4 ± 6.1 | 0.93 | 0.0007 | 1.00 | <0.0001 |
|  | White:  N (%) | 20,532 (72.1%) | | 20,532 (72.1%) | 20,533 (72.1%) | 1 | <0.0001 | 1.00 | <0.0001 |
|  | BMI (kg/m^2^), mean ± SD | 29.8 ± 7.6 | | 29.8 ± 7.6 | 29.8 ± 7.6 | 0.96 | 0.0004 | 1.00 | <0.0001 |
| ***COMORBIDITIES*** | Essential hypertension | 2795 (9.8%) | | 2797 (9.8%) | 2801 (9.8%) | 0.98 | 0.0002 | 0.97 | 0.0004 |
|  | Hyperlipidemia | 1944 (6.8%) | | 1944 (6.8%) | 1944 (6.8%) | 1 | <0.0001 | 1.00 | <0.0001 |
|  | Chronic kidney disease | 204 (0.72%) | | 221 (0.78%) | 218 (0.77%) | 0.84 | 0.0016 | 0.89 | 0.0012 |
|  | Diabetes | 1086 (3.8%) | | 1095 (3.8%) | 1096 (3.9%) | 0.41 | 0.0069 | 1 | <0.0001 |

**Supplementary Table 3: Medication Prescription Rates Over Time Including Men**

| **MEDICATION CATEGORY** |  | **TIME PERIOD** | **Nonsurgical menopause** | **Surgical Menopause** | **Men** | **p-value (Nonsurgical vs Surgical menopause)** | **p-value (Surgical menopause vs Men)** |
| --- | --- | --- | --- | --- | --- | --- | --- |
| **Statin** |  | 1 month | 0.54% | 1.16% | 1.94% | <0.0001 | <0.0001 |
|  |  | 3 months | 0.91% | 1.49% | 2.88% | <0.0001 | <0.0001 |
|  |  | 1 year | 2.08% | 2.81% | 5.12% | <0.0001 | <0.0001 |
|  |  | 3 years | 4.43% | 5.50% | 8.40% | <0.0001 | <0.0001 |
|  |  | 5 years | 5.97% | 7.17% | 10.53% | <0.0001 | <0.0001 |
| **Anti-hypertensive** |  | 1 month | 1.84% | 5.05% | 4.95% | <0.0001 | 0.56 |
|  |  | 3 months | 3.19% | 7.21% | 7.29% | <0.0001 | 0.73 |
|  |  | 1 year | 7.53% | 12.27% | 11.47% | <0.0001 | 0.0031 |
|  |  | 3 years | 13.72% | 18.53% | 16.56% | <0.0001 | <0.0001 |
|  |  | 5 years | 17.08% | 21.91% | 19.77% | <0.0001 | <0.0001 |
| **Diabetes Medications** |  | 1 month | 0.64% | 1.92% | 2.08% | <0.0001 | 0.17 |
|  |  | 3 months | 1.28% | 2.74% | 2.85% | <0.0001 | 0.42 |
|  |  | 1 year | 2.98% | 4.77% | 4.56% | <0.0001 | 0.24 |
|  |  | 3 years | 5.88% | 8.00% | 6.81% | <0.0001 | <0.0001 |
|  |  | 5 years | 7.67% | 10.06% | 8.30% | <0.0001 | <0.0001 |
|  |  |  |  |  |  |  |  |
| **ACE/ARB** |  | 1 month | 0.48% | 1.24% | 2.09% | <0.0001 | <0.0001 |
|  |  | 3 months | 0.90% | 1.71% | 3.29% | <0.0001 | <0.0001 |
|  |  | 1 year | 2.18% | 3.19% | 5.63% | <0.0001 | <0.0001 |
|  |  | 3 years | 4.04% | 5.23% | 8.71% | <0.0001 | <0.0001 |
|  |  | 5 years | 5.34% | 6.55% | 10.66% | <0.0001 | <0.0001 |
| **Beta blockers** |  | 1 month | 0.66% | 2.54% | 2.55% | <0.0001 | 0.92 |
|  |  | 3 months | 1.57% | 3.67% | 3.64% | <0.0001 | 0.86 |
|  |  | 1 year | 3.76% | 6.45% | 5.66% | <0.0001 | <0.0001 |
|  |  | 3 years | 7.15% | 9.98% | 8.35% | <0.0001 | <0.0001 |
|  |  | 5 years | 9.25% | 12.01% | 10.29% | <0.0001 | <0.0001 |
| **Calcium channel blockers** |  | 1 month | 0.32% | 0.99% | 1.28% | <0.0001 | 0.0013 |
|  |  | 3 months | 0.52% | 1.34% | 1.90% | <0.0001 | <0.0001 |
|  |  | 1 year | 1.32% | 2.31% | 3.25% | <0.0001 | <0.0001 |
|  |  | 3 years | 2.63% | 3.93% | 5.03% | <0.0001 | <0.0001 |
|  |  | 5 years | 3.55% | 4.81% | 6.31% | <0.0001 | <0.0001 |
| **Diuretics** |  | 1 month | 0.44% | 2.11% | 1.41% | <0.0001 | <0.0001 |
|  |  | 3 months | 0.96% | 2.95% | 2.17% | <0.0001 | <0.0001 |
|  |  | 1 year | 2.49% | 4.97% | 3.71% | <0.0001 | <0.0001 |
|  |  | 3 years | 4.99% | 7.75% | 5.75% | <0.0001 | <0.0001 |
|  |  | 5 years | 6.36% | 9.32% | 7.11% | <0.0001 | <0.0001 |
|  |  |  |  |  |  |  |  |
| **Metformin** |  | 1 month | 0.27% | 0.39% | 0.71% | 0.0085 | <0.0001 |
|  |  | 3 months | 0.51% | 0.63% | 1.03% | 0.0746 | <0.0001 |
|  |  | 1 year | 1.16% | 1.37% | 1.86% | 0.0222 | <0.0001 |
|  |  | 3 years | 2.30% | 2.66% | 3.15% | 0.0055 | 0.0005 |
|  |  | 5 years | 3.20% | 3.52% | 3.54% | 0.0343 | 0.95 |
| **Insulin** |  | 1 month | 0.21% | 1.55% | 1.38% | <0.0001 | 0.12 |
|  |  | 3 months | 0.43% | 2.04% | 1.82% | <0.0001 | 0.0588 |
|  |  | 1 year | 0.99% | 3.06% | 2.85% | <0.0001 | 0.14 |
|  |  | 3 years | 2.07% | 4.50% | 4.24% | <0.0001 | 0.14 |
|  |  | 5 years | 2.75% | 5.30% | 5.17% | <0.0001 | 0.49 |
| **SGLT2-i** |  | 1 month | n/a | 0.06% | 0.13% | n/a | 0.0065 |
|  |  | 3 months | 0.06% | 0.09% | 0.20% | 0.17 | 0.0009 |
|  |  | 1 year | 0.12% | 0.22% | 0.47% | 0.0046 | <0.0001 |
|  |  | 3 years | 0.31% | 0.44% | 0.84% | 0.0111 | <0.0001 |
|  |  | 5 years | 0.50% | 0.67% | 1.20% | 0.0098 | <0.0001 |
| **GLP-1** |  | 1 month | 0.18% | 0.13% | 0.17% | 0.17 | 0.33 |
|  |  | 3 months | 0.33% | 0.45% | 0.26% | 0.0222 | 0.14 |
|  |  | 1 year | 1.08% | 0.93% | 0.53% | 0.0852 | <0.0001 |
|  |  | 3 years | 2.28% | 2.27% | 1.21% | 0.93 | <0.0001 |
|  |  | 5 years | 3.23% | 3.46% | 1.70% | 0.13 | <0.0001 |
